# HER2 and PD-L1 Expression in Gastric and Gastroesophageal Junction Cancer: Insights for Combinatorial Targeting Approaches

**DOI:** 10.3390/cancers16061227

**Published:** 2024-03-20

**Authors:** Marta Baptista Freitas, Irene Gullo, Dina Leitão, Lúcia Águas, Carla Oliveira, António Polónia, Joana Gomes, Fátima Carneiro, Celso Albuquerque Reis, Henrique Oliveira Duarte

**Affiliations:** 1 Department of Medical Oncology of Unidade Local de Saúde (ULS) de São João, 4200-319 Porto, Portugal; marta.catarina.freitas@ulssjoao.min-saude.pt (M.B.F.); lucia.aguas@ulssjoao.min-saude.pt (L.Á.); 2 Department of Pathology of Unidade Local de Saúde (ULS) de São João, 4200-319 Porto, Portugal; igullo@med.up.pt (I.G.); fcarneiro@ipatimup.pt (F.C.); 3 Department of Pathology, Faculty of Medicine of the University of Porto (FMUP), 4200-329 Porto, Portugal; dinaraquel@med.up.pt; 4 Instituto de Investigação e Inovação em Saúde, Universidade do Porto (i3S), 4200-135 Porto, Portugal; carlaol@i3s.up.pt (C.O.); apolonia@ipatimup.pt (A.P.); joanag@ipatimup.pt (J.G.); celsor@ipatimup.pt (C.A.R.); 5 Faculty of Medicine of the University of Porto (FMUP), 4200-319 Porto, Portugal; 6 Institute of Molecular Pathology and Immunology of the University of Porto (IPATIMUP), 4200-135 Porto, Portugal; 7 Instituto de Investigação, Inovação e Desenvolvimento, Fundação Fernando Pessoa (FP-I3ID), 4249-004 Porto, Portugal; 8 Instituto de Ciências Biomédicas Abel Salazar (ICBAS), Universidade do Porto, 4050-313 Porto, Portugal

**Keywords:** gastric adenocarcinoma, gastroesophageal junction adenocarcinoma, Human Epidermal Growth Factor Receptor 2 (HER2), programmed death-ligand 1 (PD-L1), targeted therapy

## Abstract

**Simple Summary:**

In advanced, unresectable and metastatic gastric and gastroesophageal junction adenocarcinoma (GA/GEJA), Human Epidermal Growth Factor Receptor 2 (HER2) and programmed death-ligand 1 (PD-L1) expression play an important role in treatment selection. However, their potential clinical application in earlier stages of disease remains unexplored. The aim of this study was to evaluate the expression patterns of HER2 and PD-L1 in a curative-intent GA/GEJA cohort. Furthermore, we analyzed the association between HER2 expression and clinicopathological features. Among 107 patients, 8.4% were HER2-positive and seven of these also had a PD-L1 combined positive score of ≥1. HER2 status was not statistically significantly associated with survival outcomes. A pathologist-guided, region-specific analysis revealed that PD-L1 expression rarely overlaps with HER2-positive tumor areas. These novel findings indicate that combinatorial strategies targeting HER2 and PD-L1 might be directed toward distinct tumor subclones. These different biomarker expression patterns may have important therapeutic and prognostic implications.

**Abstract:**

Gastric and gastroesophageal junction adenocarcinomas (GA/GEJA) are associated with a poor prognosis, primarily due to late disease diagnosis. Human Epidermal Growth Factor Receptor 2 (HER2) overexpression and programmed death-ligand 1 (PD-L1) expression are important biomarkers for treatment selection in locally advanced unresectable and metastatic GA/GEJA, and there is increasing interest in their role in earlier stages of disease. In this study, we aimed to evaluate HER2 and PD-L1 expression in a curative-intent GA/GEJA cohort to describe their expression patterns and analyze the association between HER2 expression and clinicopathological features. HER2 expression was evaluated in surgical and endoscopic submucosal dissection tumor samples, and PD-L1 was evaluated in HER2-positive cases. The clinical cohort included 107 patients, with 8.4% testing positive for HER2 (seven of whom also exhibited a PD-L1 combined positive score of ≥1. HER2 status was not significantly associated with survival outcomes. A pathologist-guided, region-specific analysis revealed that PD-L1 expression rarely overlaps with HER2-positive tumor areas. While the therapeutic implications of these observations remain unknown, these findings suggest that combination strategies targeting HER2 and PD-L1 might be directed toward distinct tumor subclones. The herein disclosed region-specific biomarker expression patterns may have important therapeutic and prognostic impacts, warranting further evaluation.

## 1. Introduction

Gastric cancer is the fifth most common cancer type and the fourth leading cause of cancer death worldwide [1]. In recent decades, a decrease in gastric cancer incidence has been seen in high-risk countries of Western Europe and North America [2]. In the United States, gastric cancer has a substantially higher incidence and mortality rate among Black and Native American individuals compared to the Caucasian population [3]. On the other hand, the incidence of gastroesophageal junction cancer has been increasing in North America in recent years [4]. Both gastroesophageal and gastric cancer are associated with poor prognosis, mainly due to late diagnosis [5]. In the advanced gastric cancer setting, 5-year survival is less than 30% [6]. The majority of gastric cancer cases are adenocarcinomas (more than 95%) and are classified according to their anatomic location (cardia/proximal or non-cardia/middle and distal) and histological subtype, according to Laurén and World Health Organization (WHO) classifications [5,6]. For early gastric adenocarcinoma (GA), in which tumor invasion is no deeper than the submucosa, endoscopic submucosal dissection (ESD) can be the selected therapeutic strategy [2,6]. For stage IB-III disease, surgery plus D2 lymphadenectomy is a potentially curative treatment, although most patients experience disease relapse after resection [2]. Thus, combined therapeutic modalities are the standard of care for these stages: peri-operative chemotherapy (POCT) plus surgery or, in cases of surgery without pre-operative CT, adjuvant CT should be given after surgery [2]. Regarding locally advanced GEJA, patients should undergo POCT or neoadjuvant chemoradiotherapy (NACRT) followed by surgery (plus adjuvant nivolumab in the case of residual pathologic disease in the resected specimen) [4].

For locally advanced unresectable and metastatic GA/GEJA, predictive biomarkers guiding treatment decisions include Human Epidermal Growth Factor Receptor 2 (HER2/c-ErbB2) expression/genomic amplification, programmed death-ligand 1 (PD-L1) expression, and microsatellite instability (MSI)/mismatch repair deficiency (MMRd) [7]. The cell surface overexpression of the HER2 receptor tyrosine kinase, which occurs mainly due to the genomic amplification of the *ERBB2* oncogene, has been associated with GEJA and GA development, but the prognostic meaning of HER2 status remains unclear [5,8]. HER2 positivity, defined as a HER2 immunohistochemistry (IHC) score of 3+ or HER2 IHC 2+ plus ISH (in situ hybridization) positivity, varies widely, being around 2–45% in GEJA and 10–20% in GA [2,8]. Moreover, HER2 overexpression is more common in intestinal than in the diffuse GA subtypes [5]. In addition, HER2 overexpression in GA has shown spatial (intratumor) and temporal molecular heterogeneity, which severely hinders the pathology-guided assessment of the HER2 status and the cost-effective allocation of GA patients towards anti-HER2 therapeutic regimens [9]. Despite HER2 heterogeneity, the ToGA clinical trial showed significant median overall survival (OS) improvement in HER2-positive advanced unresectable GA/GEJA receiving CT (cisplatin and a fluoropyrimidine) in combination with trastuzumab (humanized monoclonal antibody (mAb) against the extracellular region of HER2) comparing to CT alone (13.8 vs. 11 months, respectively; *p* = 0.046) [10]. This phase III trial established a combination of platin plus fluoropyrimidine CT with trastuzumab as the standard treatment for HER2-positive metastatic GEJA and GA [5,10].

PD-L1 binds to its cognate receptor Programmed Cell Death Protein 1 (PD-1) on T cells recruited to the tumor microenvironment preventing T cell activation and leading to tumor immune escape [11]. Higher PD-L1 expression in tumor cells and tumor-infiltrating lymphocytes (TILs) has been associated with worse outcomes in patients with localized GEJA [11]. Regarding PD-L1 testing, IHC for the evaluation of PD-L1 expression should be performed on locally advanced, recurrent, or metastatic GA/GEJA, which are candidates to undergo treatment with PD-1 inhibitors [2,5,8]. One of the scoring systems for PD-L1 evaluation is the combined positive score (CPS), defined as the number of PD-L1-positive cells (tumor cells, TILs, and macrophages) divided by the total number of viable tumor cells, multiplied by 100 [11]. Around 50% to 60% of GA are PD-L1 CPS ≥ 1 [2]. The level of PD-L1 expression is a predictive biomarker of therapeutic response to immune checkpoint inhibitors (ICIs) [12]. The CheckMate 649 trial showed a significant OS benefit in patients with HER2-negative, PD-L1 CPS ≥ 5, unresectable GA/GEJA treated with nivolumab (anti-PD-1 mAb) plus CT in comparison to CT alone (13.1 vs. 11.1 months, respectively; *p* < 0.0001) [13]. In this setting, pembrolizumab (anti-PD-1 mAb) in combination with platinum-based CT also showed an OS benefit in patients with GA/GEJA PD-L1 CPS ≥ 1 when compared to placebo plus CT in the KEYNOTE-859 trial (13.1 vs. 11.4 months, respectively; *p* < 0.0001) [14].

Recent studies evaluating the combination of anti-HER2 therapeutics with ICIs in advanced GA/GEJA have produced promising results. Interestingly, HER2 signaling is associated with the recruitment and activation of tumor-infiltrating immune cells [12]. These studies have shown that trastuzumab therapy may upregulate PD-1 and PD-L1 expression and that, conversely, ICIs may improve the therapeutic efficacy of anti-HER2 agents [12]. Based on these results, phase I and II clinical trials have shown the treatment efficacy of trastuzumab and ICIs in combination with CT in HER2-positive GA/GEJA [15]. Since May 2021, the Food and Drug Administration (FDA) granted the accelerated approval for the addition of pembrolizumab to trastuzumab and CT (with platinum and fluoropyrimidine) in locally advanced unresectable or metastatic HER2-positive GA/GEJA [2,5,8]. This approval was based on the interim analysis of KEYNOTE-811, which showed a significant improvement in overall response rates in the combination arm (74.4% vs. 51.9%, *p* = 0.00006) [16,17]. A new interim analysis on survival outcomes showed a median progression-free survival (PFS) for the pembrolizumab–trastuzumab–CT arm of 10.8 months vs. 7.2 months (*p* = 0.0001) and an OS of 20.5 months vs. 15.6 months (*p* = 0.0143) in patients with HER2-positive PD-L1 CPS ≥ 1 tumors [16]. These results lead to the approval of this combination by the European authorities and to indication restriction by FDA (approved only for patients with tumors PD-L1 CPS ≥ 1) [18,19].

GA/GEJA are significant global health burdens, with poor prognosis primarily due to late-stage diagnosis. HER2 overexpression and PD-L1 expression are crucial biomarkers for treatment selection in advanced GA/GEJA. However, the role and clinical application of HER2 and PD-L1 in earlier stages warrants further investigation. Moreover, currently available therapeutic options targeting specific molecular alterations, such as trastuzumab, are challenged by tumor molecular heterogeneity and the widespread emergence of acquired or innate molecular resistance. Therefore, this study aimed to evaluate the expression patterns of HER2 and PD-L1 in a curative-intent GA/GEJA cohort. Additionally, we analyzed the association between HER2 expression and clinicopathological features. By investigating these biomarkers in earlier stages, we have produced valuable insights into their potential prognostic and therapeutic implications.

## 2. Materials and Methods

### 2.1. Study Design and Clinical Sample Collection

Adult patients with a histologically confirmed diagnosis of GA and GEJA, treated with curative intent between January 2015 and December 2017 at Unidade Local de Saúde (ULS) de São João, Porto, Portugal, were retrospectively selected. Exclusion criteria included gastric or gastroesophageal tumors other than adenocarcinoma, no available tumor tissue sample adequate for HER2 and PD-L1 biomarker evaluation, or the absence of follow-up data. Tumors were re-staged according to the American Joint Committee on Cancer (AJCC) TNM staging, eighth edition, 2017 [20]. Our study included tumor samples from “chemo-naive” patients and patients submitted to NACRT or POCT. Tumor response grade (TRG) of GA/GEJA following neoadjuvant therapy was classified following the grading system proposed by Becker et al. [21]. In all the selected patients, tumor tissue samples for biomarker evaluation were obtained in surgery (upfront surgery or after POCT/NACRT) and ESD. For every GA/GEJA patient included in the cohort, two distinct formalin-fixed paraffin-embedded (FFPE) tissue blocks were selected for the evaluation and scoring of HER2 expression, to account for the significant spatial heterogeneity commonly observed for HER2. PD-L1 was evaluated in HER2-positive tumors (score of 3+ or score of 2+ with ISH+) and in HER2-negative cases (score 0) with minor components (<10%) showing HER2 expression 2+/3+.

The study was conducted in accordance with the Declaration of Helsinki and the protocol was approved by the Ethical Committee of ULS de São João (reference CES 236-14).

### 2.2. Immunohistochemical Staining of HER2 and PD-L1

From each selected FFPE tissue block, serial 3 μm tissue sections were prepared. Immunohistochemical staining was performed in an automated Ventana BenchMark ULTRAStaining System, using OptiView DAB IHC Detection Kit (Roche/Ventana Medical Systems, Tucson, AZ, USA), according to the manufacturer’s instructions. HER2 immunostaining was performed using the ready-to-use anti-HER2 4B5 antibody clone (Roche), following an antigen retrieval step using pH = 6.0 acidic citrate buffer. PD-L1 staining was performed using the clone 22C3, 1:100 (Dako, Agilent Technologies, Santa Clara, CA, USA). For each stained tissue slide, internal positive and negative controls for each antibody were included.

HER2 and PD-L1 staining were evaluated and scored by a board-certified pathologist with experience in gastric pathology, following approved biomarker-specific guidelines [22,23].

### 2.3. In Situ Hybridization and Interpretation

ISH was performed on 3 μm tissue sections in one block of each case with dual-hapten, dual-color ISH (DDISH). The FDA-approved dual-probe assay (VENTANA HER2 Dual ISH DNA Probe Cocktail, Ventana Medical Systems) contains a HER2 locus-specific probe (black signal) and a control probe specific for the centromere of chromosome 17 (centromere enumeration probe-CEP17, red signal), which allows for the detection of HER2 gene amplification using light microscopy. The entire procedure was carried out on an automated staining system (Ventana BenchMark XT Staining System; Ventana Medical Systems) according to the manufacturer’s instructions. Adequate positive and negative controls were used in every set of slides. The samples were classified by a pathologist, according to the 2016 HER2 guideline for GEA and the 2016 HER2 guideline for GA [22]. Corresponding hematoxylin and eosin (H&E) staining was used to identify the invasive component of the tumor, and, whenever available, the HER2 IHC slide was used to score the area with strongest intensity. Only cells with a minimum of one copy of HER2 and CEP17 each were scored. The number of HER2 signals was estimated in clusters, except for doublets, which were counted as a single signal. The evaluation of the samples included a scoring of at least 20 nuclei, in two different areas, recording the numbers of HER2 and CEP17 signals over an area with a higher level of HER2 gene amplification. The 2016 GEA guideline was used for result interpretation, and HER2 gene amplification was classified as positive when the HER2/CEP17 ratio is ≥2.0 or <2.0 and the average HER2 copy number is ≥6.0 signals per cell, and negative when the HER2/CEP17 ratio is <2.0 and the average HER2 copy number is <6.0 signals per cell [22].

### 2.4. Statistical Analysis

Clinicopathological features, treatment, and follow-up data from GA/GEJA patients were collected. Patient OS was calculated from the time of biopsy-confirmed diagnosis to the date of death or last clinical follow-up. Categorical variables were described as absolute and relative frequencies and continuous variables were described using median values. Categorical variables were compared using the Chi-square test or Fisher exact test, as appropriate. Kaplan–Meier estimates of OS and univariate analysis were carried out using the Log Rank test. Multivariate analysis was performed using Cox Regression. Differences were considered statistically significant if *p* < 0.05. Statistical analyses were performed using Statistical Package for the Social Sciences (SPSS, IBM Inc., New York, NY, USA) version 25.

## 3. Results

### 3.1. Overall Characteristics and Clinical Factors

The studied clinical series encompassed 107 patients, with 70 males (65.4%) and a median age of 68 (26–93) years, mostly diagnosed with early-stage disease (cTNM stage I and II: *n* = 72; 67.3%). Two patients were diagnosed with oligometastatic disease, which, after discussion at the Multidisciplinary Tumor Board (MTB), were treated with curative intent (POCT followed by gastric surgery and metastasectomy). Twelve patients (11.2%) harbored GEJA, seven of these were GEJA Siewert 1 and 2 and were treated with NACRT followed by surgery. Regarding the stomach, the most common tumor locations were the antrum and pylorus (62.6%). Most of the adenocarcinomas had tubular and/or papillary histology by WHO classification (44.9%), intestinal subtype by Laurén classification (43%), and showed an infiltrative growth pattern (76.6%). More than half of the patients (68.2%) were submitted to upfront surgery and 22.4% patients underwent POCT. Clinicopathological and treatment characteristics of patients included in this study are described in Table 1.

### 3.2. HER2 and PD-L1 Status

Nine patients (8.4%) were classified as HER2-positive (HER2 3+ or HER2 2+ with positive dual ISH) (Table 2). In the three inconclusive HER2 2+ cases, dual ISH was performed and all confirmed HER2 amplification.

When clinicopathological features of patients harboring HER2-positive (2+/3+) or HER2-negative (0/1+) GA/GEJA were compared, no clinicopathological feature was statically significantly associated with HER2 status (Table 3).

To evaluate whether HER2-positive patients could be potential responders to ICIs, PD-L1 status was assessed in those tumors and in two cases classified as HER2-negative (score 0), yet with a minor component (<10%) showing HER2 expression (2+/3+) (Table 3). In the nine HER2-positive cases, seven (77.8%) were PD-L1 CPS ≥ 1 (Table 4).

### 3.3. HER2 and PD-L1 Expression Overlapping in Tumors

**Figure 1:**
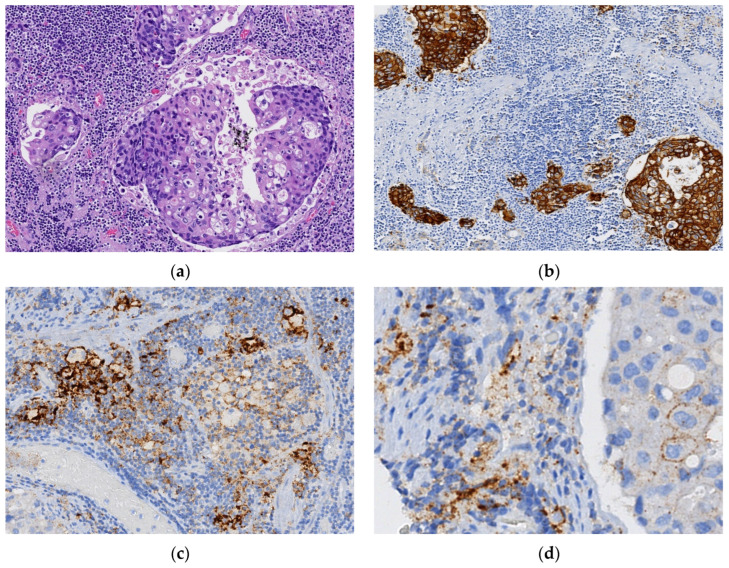
Gastric adenocarcinoma (GA) tubular solid subtype by WHO ((**a**): hematoxylin and eosin (H&E) staining, 200× magnification) with overlapping expression of HER2 (IHC 3+) (**b**): 200× magnification) and PD-L1 (**c**): 200× magnification; (**d**): tumoral cell membrane with PD-L1 expression, 400× magnification).

In seven of the nine HER2-positive cases in which PD-L1 expression was evaluated, there was no observed biomarker staining overlap in PD-L1 and HER2 co-expressing specimens, i.e., PD-L1 and HER2 expression patterns were mutually exclusive, including in the two cases in which PD-L1 CPS was <1 (Table 4). Only two cases, one treated with upfront surgery and another with NACRT, showed tumor regions with predominantly overlapping expression of HER2 and PD-L1. Figure 1 illustrates a surgical specimen from a HER2-positive tumor with PD-L1 CPS ≥ 5, depicting the coincident and overlapping expression of both biomarkers in the same tumoral area.

**Figure 2:**
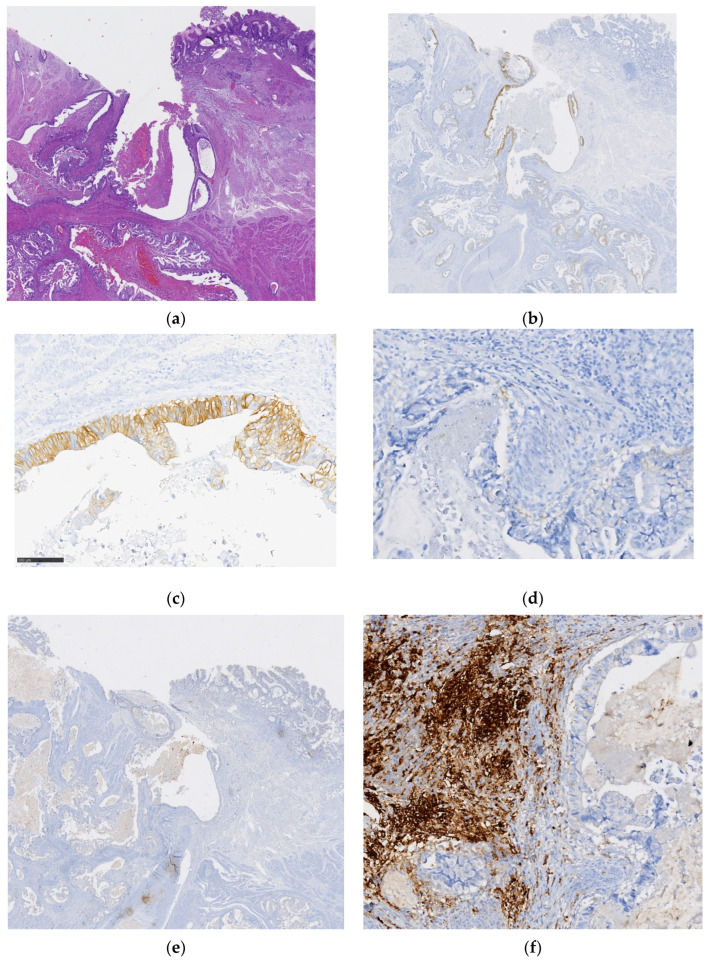
Gastric adenocarcinoma (GA) tubular/papillary subtype by WHO classification ((**a**): hematoxylin and eosin (H&E) staining, low magnification), with overall HER2 score 0 and a minor component (<10%) showing HER2 expression (2+/3+) ((**b**): HER2 expression, low magnification; (**c**): HER2 overexpression, region 1, 200× magnification). PD-L1 expression was observed in tumor areas without HER2 expression ((**d**): HER2-negative area, region 2, 200× magnification; (**e**): PD-L1 expression in region 2, low magnification; (**f**): PD-L1 expression in region 2, 200× magnification).

In the two cases classified as HER2-negative (score 0), but with a minor component (<10%) showing HER2 expression (2+/3+), PD-L1 revealed a CPS of 4 and 5 (one patient was submitted to upfront surgery and the other to POCT, respectively). In both cases, the tumor areas with HER2 overexpression were not coincident with the PD-L1-positive areas. Figure 2 illustrates one such case, with globally negative HER2 but a minor component (<10%) with HER2 expression (3+) and PD-L1 CPS ≥ 5, revealing histological regions of the tumor component depicting mutually exclusive expressions of HER2 and PD-L1 biomarkers.

### 3.4. Patient Outcomes

Median follow up time was 45 (1–107) months. Thirty-five (32.7%) patients had disease relapse or progression, and thirty-one (29%) had distant metastases. Forty-eight-month PFS was 63.5% (median PFS not reached). During follow-up time, 64 (59.8%) patients died, 32 (29.9%) due to GA/GEJA. Median OS was 44 months (CI 95%, 21.70–66.29). Patients with lower clinical- (cTNM = I or II) or pathological (pTNM = I or II)-stage tumors had better OS than those with higher-stage tumors (cTNM or pTNM III–IV): 81 months (CI 95%, 57.46–104.53) vs. 26 months (CI 95%, 12.91–39.09), *p* < 0.01; 88 months (CI 95%, 66.37–109.62) vs. 24 months (CI 95%,14.47–33.53), *p* < 0.01, respectively. Tumors with mucosa, submucosa or muscular layer invasion (pT1 and pT2) were significantly associated with better OS compared to tumors invading subserosa or serosa (pT3 and pT4) (81 months (IC 95%, 56.07–105.92) vs. 28 months (IC 95%, 13.33–42.67), *p* = 0.02. Patients without positive lymph nodes (pN-) had significantly better OS than those with lymph node metastases (pN+): 48-month OS of 20.4% vs. 47.7%, *p* < 0.01. The absence of distant metastasis following surgery (pM) contributed to better OS: 61 months (CI 95%, 29.28–92.72) vs. 16 months (CI 95%, 5.15–26.85), *p* < 0.01. Negative surgical margins (R0) were significantly associated with better OS than positive (R+) or undetermined (Rx): 48 months (IC 95%, 12.14–83.89) vs. 23 months (IC 95%, 4.66–41.34), *p* = 0.02. Patients who underwent POCT or NACRT treatment also had better OS than those who did not complete POCT/NACRT treatment: 66 months (IC 95%, 13.66–118.34) vs. 19 months (IC 95%, 10.68–27.32), *p* = 0.03. Tumor lymphatic permeation and perineural invasion were both associated with worse OS: 42-month OS of 42.1% vs. 49.4%, *p* = 0.01; 36-month OS of 24.4% vs. 51.2%, *p* < 0.01, respectively. HER2 status did not show a significant association with OS: 36-month OS of 57.7% for HER2-positive patients vs. 33.3% for HER2-negative patients, *p* = 0.32. Associations between clinicopathological features and OS are detailed in Table 5. Multivariate analysis confirmed the association between lymph nodes metastases (pN+) and worse OS (HR 0.06, IC 95%, 0.01–0.48, *p* = 0.01) (Table 6).

## 4. Discussion

Gastric-cancer-related mortality and therapeutic management remain economic and clinical burdens on a global scale. The histological and molecular heterogeneity inherent to GA/GEJA impair the allocation of individual patients towards well-defined therapeutic modalities, and undermines the quest for novel therapeutic agents capable of overcoming molecular mechanisms of resistance [24]. In recent years, efforts have been made towards the comprehensive molecular profiling of gastric tumors at the multi-omic level, and novel classification systems have been proposed, in which GA are grouped according to their molecular features, including their biological dependency on specific oncogenic signaling pathways, and their differential eligibility towards targeted therapeutic modalities [25,26].

Indeed, recently proposed molecular subgroups of GA have been associated with patients’ clinical outcome and therapeutic response [26]. In the locally advanced unresectable/metastatic GA/GEJA clinical setting, HER2 overexpression/amplification, MSI/MMRd and PD-L1 expression remain the only biomarkers determining patient eligibility towards anti-HER2- and ICI-based targeted therapies in the clinical practice, following ToGA (testing trastuzumab), CheckMate 649 (using nivolumab), KEYNOTE-859 (with pembrolizumab) clinical trials for first-line treatments [7,10,13,14,18]. Despite moderate improvement in the OS of treated patients, both trastuzumab and anti-PD-1 therapies (nivolumab and pembrolizumab), targeting cell surface HER2 on cancer cells and PD-1 on immune cells of the tumor microenvironment, respectively, bear limited clinical efficacy due to the widespread emergence of tumor resistance [24]. Recently, following KEYNOTE-811, the FDA and European Medicines Agency (EMA) granted accelerated approval for the therapeutic combination of pembrolizumab and trastuzumab in patients bearing HER2-positive and PD-L1 CPS ≥ 1 GA/GEJA, who had not received prior systemic chemotherapy for metastatic disease [18,19].

In the present study, we assessed the prospective eligibility of GA/GEJA patients to potentially benefit from the recently approved combination therapy of pembrolizumab–trastuzumab–CT, in a clinical surgical cohort (earlier stages than those included in the clinical trial testing the combination), through the tissue-based expression of both HER2- and PD-L1-predictive biomarkers. Importantly, the surgical cohort described herein is enriched for early disease stages (I–III), since only complete surgical specimens, obtained from either total or partial gastrectomy or ESD, in the case of early-stage confined lesions, were analyzed. Two of the included patients were intraoperatively diagnosed with oligometastatic clinical stage IV GA and treated with curative intent (POCT, gastrectomy, and metastasectomy). Upon evaluation of the surgical specimens, eleven cases were classified as pathological stage IV disease, due to the presence of microscopical peritoneal implants. Nevertheless, they had been submitted to a curative-intent surgery. In the cases submitted to NACRT or POCT therapy, PD-L1 and HER2 expression was evaluated in the surgical specimen (obtained after preoperative CT or CRT), which may modify the expression levels and heterogeneity patterns of both biomarkers. Regarding HER2 expression, six patients had a HER2 score of 3+, required for trastuzumab eligibility. Three patients received the equivocal HER2 score classification of 2+, but were later confirmed to have *ERBB2* genomic amplification, and thus confirmed as HER2-positive by dual ISH. Therefore, nine patients out of 107 (8.4%) were considered HER2-positive. This result is in line with what is reported in the literature, in which the HER2 positivity rate in GA clinical cohorts varies widely, between 7–34% [10]. This observation can be further sustained by the significant lack of stage IV patients in our series. Interestingly, there was a tendency for higher HER2 expression in well-differentiated tubular/papillary WHO tumor subtypes (*n* = 7/9), as well as in the intestinal sybtype by Laurén (*n* = 6/9). Although these findings were not statistically significant, they are in accordance to previous reports [27].

Regarding survival outcomes, HER2 expression was not correlated with patient OS, as previously reported for early-stage GA/GEJA [27]. Other factors related to early-stage, non-metastatic disease (cTNM I-II, pTNM I-II, pT1, pN0, pM0, R0 surgery, absence of tumor lymphatic permeation, and perineural invasion) are shown to be correlated with significantly improved OS. These findings are based on well-documented worse survival outcomes among patients with advanced stage gastric cancer [28]. In locally advanced stages, POCT showed a significant improvement in survival outcomes [8,28]. In our cohort, locally advanced adenocarcinomas located in GEJA Siewert 1 or 2 were treated with NACRT followed by surgery, while those located in the stomach were treated with POCT and surgery, unless the patient was unfit for CT or had an emergency situation requiring upfront surgery. The conclusion of NACRT or POCT was also a significant prognostic factor in this cohort, as expected from previous studies [28]. Additionally, GA/GEJA patients should be treated at high-volume centers such as ours, as this also bears prognostic impact [29].

The expression of HER2 in both gastric and gastroesophageal junction cancer tissue specimens is characterized by high levels of heterogeneity. Indeed, both spatial (intratumoral) and temporal heterogeneity in HER2 expression levels are considered to be one of the main causes underlying the failure or underperformance of clinical trials evaluating HER2-targeting therapeutic modalities. The continuous therapeutic pressure by HER2-directed agents might lead to the selective elimination of HER2-positive tumor clones, while favoring the expansion of low-HER2 or HER2-negative clones. Furthermore, the prolonged exposure of gastric neoplasms to trastuzumab-based regimens favors the temporal loss of HER2 overexpression, and the concomitant upregulation of bypass signaling circuits sustaining tumor cell proliferation and survival (e.g., EGFR and c-Met-mediated signaling) [24]. The binding specificity of trastuzumab, rather than its therapeutic performance, has been exploited in the context of drug delivery, namely in the format of antibody-drug conjugates, opening promising new therapeutic avenues for the clinical management of low-HER2-expressing tumors [24]. In the analyzed HER2-positive cases, significant spatial heterogeneity in HER2-positive regions was observed. In our cohort, seven out of nine (77.8%) HER2-positive tumors were also PD-L1 CPS ≥ 1. Previous studies showed higher and lower rates of PD-L1 CPS ≥ 1 in HER2-positive tumors, which can be related to population heterogeneity or differences in biomarker evaluation, and should be further explored [30,31]. Interestingly, previously proposed molecular classification systems of gastric cancer do not place HER2 and PD-L1 enrichment within the same tumor subtypes [26]. Although the efficacy of the combination of pembrolizumab–trastuzumab–CT in earlier GA/GEJA stages has not yet been established, these seven patients would be potentially eligible to undergo this combination therapy in a metastatic setting [18]. Interestingly, a pathologist-guided, region-specific analysis revealed that PD-L1 expression rarely overlaps with HER2-positive areas, suggesting that PD-L1 and HER2 biomarkers may bear mutually exclusive expression patterns in tumor specimens. The present study provided valuable insights regarding the expression patterns of HER2 and PD-L1 in a curative-intent GA/GEJA cohort. Notably, our findings revealed that PD-L1 expression rarely overlapped with HER2-positive tumor areas, suggesting the existence of distinct tumor subclones. Although the therapeutic implications of these observations remain unknown, they highlight the possibility of combinatorial strategies targeting HER2 and PD-L1, which may need to be tailored to specific tumor molecular profiles. Indeed, an interim analysis of the KEYNOTE-811 trial showed a survival benefit with the combined therapy (pembrolizumab–trastuzumab–CT) in HER2-positive and PD-L1 CPS ≥ 1 metastatic GA/GEJA [18,19]. Whether the clinical efficacy of the pembrolizumab plus trastuzumab combination therapy is influenced by the degree of HER2 and PD-L1 overlapping expression has not been addressed in this study and warrants further investigation. Biomarker-guiding therapy is showing promising results in GA/GEJA with increasing clinical practice applicability, mainly in the metastatic setting [5,8,18]. These observations on HER2 and PD-L1 expression patterns may bear a significant therapeutic and prognostic impact.

## 5. Conclusions

The findings of a non-overlapping expression of HER2 and PD-L1 biomarkers in a GA/GEJA cohort may suggest that combinatorial strategies targeting HER2 and PD-L1 might be directed to distinct tumor subclones. However, the therapeutic and prognostic implications of these findings remain unknown and should be further evaluated in future clinical trials.

## Figures and Tables

**Table 1:** Clinicopathological characteristics and therapy information of patients with gastric or gastroesophageal junction adenocarcinoma (GA/GEJA).

Clinicopathological Parameters *n* = 107		*n* (%)
Sex	Male	70 (65.4)
	Female	37 (34.6)
Age	<65 years	44 (41.1)
	≥65 years	63 (58.9)
Cardiac disease		21 (19.6)
Cerebrovascular disease		4 (3.7)
Clinical stage (cTNM) *	I	51 (47.7)
	II	21 (19.6)
	III	30 (28.0)
	IV	2 (1.9)
	Unclassified	3 (2.8)
Tumor anatomical location	GEJA, Siewert 1–2	7 (6.5)
	GEJA, Siewert 3	5 (4.7)
	Gastric, Fundus	5 (4.7)
	Gastric, Body	14 (13.1)
	Gastric, Body and Antrum	6 (5.6)
	Gastric, Antrum/Pylorus	67 (62.6)
	Gastric, All stomach	3 (2.8)
Histological WHO classification	Tubular and/or Papillary	48 (44.9)
	Mucinous	3 (2.8)
	Poorly Cohesive	18 (16.8)
	Mixed	35 (32.7)
	Unclassified	3 (2.8)
Histological Laurén classification	Intestinal	47 (43.0)
	Diffuse	13 (12.1)
	Indeterminate type	45 (42.1)
	Unclassified	3 (2.8)
Growth pattern	Infiltrative	82 (76.6)
	Expansive	15 (14.0)
	Unclassified	10 (9.4)
Depth of invasion (pT)	T1	32 (29.9)
	T2	16 (15.0)
	T3 and T4a	58 (54.2)
	T4b	0
	Unclassified	1 (0.9)
Lymphatic permeation	Present	60 (56.1)
	Absent	44 (41.1)
	Unclassified	3 (2.8)
Perineural invasion	Present	43 (40.2)
	Absent	61 (57.0)
	Unclassified	3 (2.8)
Vascular invasion	Present	42 (39.3)
	Absent	62 (57.9)
	Unclassified	3 (2.8)
Surgical margins	R0	96 (89.7)
	R+ (R1–R2)	8 (7.5)
	Rx	3 (2.8)
LN metastasis (pN)	Present	55 (51.4)
	Absent	49 (45.8)
	Unclassified	3 (2.8)
Positive LN	Median (min–max)	1 (0–37)
Total number of removed LN	Median (min–max)	23 (0–61)
Positive LN—Total number of removed LN ratio	Median (min–max)	0.05 (0–1)
Distant metastasis after surgery (pM)	Present	11 (10.3)
	Absent	95 (88.8)
Pathological staging (pTNM) *	I	38 (35.5)
	II	25 (23.4)
	III	31 (29.0)
	IV	13 (12.1)
Upfront treatment approach	ESD	3 (2.8)
	Surgery	73 (68.2)
	POCT	24 (22.4)
	NACRT	7 (6.5)
POCT conclusion (*n* = 24)		11 (45.8)
Tumor response grade after POCT (*n* = 24) **	1b	5 (20.8)
	2	7 (29.2)
	3	12 (50.0)
NACRT conclusion (*n* = 7)		2 (28.6)
Tumor response grade after NACRT (*n* = 7) **	1a	1 (14.4)
	2	2 (28.6)
	3	3 (42.6)
	Unknown	1 (14.4)
ACT (*n* = 76 ***)		22 (28.9)
ACT conclusion (*n* = 22)		16 (72.7)

Legend: ACT: adjuvant chemotherapy, ESD: endoscopic submucosal dissection, GEJA: gastroesophageal junction adenocarcinoma, LN: lymph nodes, NACRT: neoadjuvant chemotherapy concomitant to radiotherapy, POCT: perioperative chemotherapy, RT: radiotherapy, WHO: World Health Organization. * Eighth edition American Joint Committee on Cancer, ** Becker Classification, *** number of patient eligible to ACT (excluded those who did POCT and NACRT).

**Table 2:** Characterization of HER2 and PD-L1 expression in gastric and gastroesophageal junction adenocarcinomas (GA/GEJA).

Biomarker Expression, *n* = 107		*n* (%)
HER2 status	0	97 (90.6)
	1+	1 (0.9)
	2+ ISH+	3 (2.8)
	3+	6 (5.6)
PD-L1 CPS	Non-tested	96 (89.7)
	<1	2 (1.9)
	1	3 (2.8)
	4	1 (0.9)
	5	2 (1.9)
	7	1 (0.9)
	8	1 (0.9)
	10	1 (0.9)

Legend—CPS: combined score, ISH: in situ hybridization.

**Table 3:** Association analysis between clinicopathological/therapy features and HER2 expression.

Clinicopathological Parameters *n* = 107		HER2 Negative (*n* = 98)	HER2 Positive (*n* = 9)	*p* Value
Sex	Male	63	7	0.34
	Female	35	2	
Age	<65 years	40	4	0.55
	≥65 years	58	5	
Cardiac disease	Yes	18	3	0.28
	No	73	6	
Cerebrovascular disease	Yes	3	1	0.32
	No	88	8	
Clinical stage (cTNM) *	I–II	66	6	0.56
	III–IV	29	3	
Tumor anatomical location	GEJA	11	1	0.41
	Gastric, Antrum/Pylorus	63	4	
	Gastric, Other	24	4	
Histological WHO classification	Tubular/Papillary	41	7	0.11
	Mixed	34	1	
	Mucinous	3	0	
	Poorly Cohesive	18	0	
Histological Laurén classification	Intestinal	40	6	0.28
	Diffuse	13	0	
	Indeterminate type	42	3	
Growth pattern	Infiltrative	76	6	0.24
	Other	17	3	
Depth of invasion (pT)	T1 + T2	43	5	0.38
	T3 + T4a	54	4	
Lymphatic permeation	Present	54	6	0.42
	Absent	41	3	
Perineural invasion	Present	41	2	0.20
	Absent	54	7	
Vascular invasion	Present	38	4	0.53
	Absent	57	5	
Surgical margins—R0	Yes	87	9	0.36
	No	11	0	
Lymph node metastasis (pN)	Present	50	5	0.57
	Absent	45	4	
Distant metastasis after surgery (pM)	Present	11	0	0.36
	Absent	86	9	
Pathological stage (pTNM) *	I-II	57	6	0.45
	III-IV	41	3	
Upfront treatment approach	ESD or Surgery	72	4	0.08
	POCT or NACRT	26	5	
POCT	Yes	20	4	0.11
	No	78	5	
Tumor response grade after POCT (*n* = 24) **	1b	4	1	0.51
	2	5	2	
	3	11	1	
NACRT	Yes	6	1	0.47
	No	92	8	
Tumor response grade after NACRT (*n* = 7) **	1–2	2	1	0.50
	3	3	0	
POCT/NACRT conclusion (*n* = 31)	Yes	10	3	0.34
	No	16	2	
ACT (*n* = 76 ***)	Yes	22	0	0.25
	No	50	4	
Disease relapse/progression	Yes	32	3	0.61
	No	66	6	

Legend: ACT: adjuvant chemotherapy, ESD: endoscopic submucosal dissection, GEJA: gastroesophageal junction adenocarcinoma, NACRT: neoadjuvant chemotherapy concomitant to radiotherapy, POCT: perioperative chemotherapy, WHO: World Health Organization. * Eight edition American Joint Committee on Cancer, ** Becker Classification, *** number of patient eligible to ACT (excluded those who did POCT and NACRT).

**Table 4:** PD-L1 expression pattern in HER2-positive cases.

Case ID	Upfront Treatment Approach	HER2 Expression	CPS PD-L1		
			<1	≥1–<5	≥5
32	POCT	3+			
46	POCT	3+			
56	Sg	3+			
68	POCT	3+			
105	Sg	3+			
111	NACRT	3+			
59	POCT	2+			
69	Sg	2+			
98	Sg	2+			

Legend: Orange: overlapping HER2 and PD-L1 expression; Blue: non-overlapping HER2 and PD-L1 expression. POCT: perioperative chemotherapy; Sg: upfront surgery.

**Table 5:** Association between clinicopathological/therapy features with overall survival (OS) (univariate analysis).

Clinicopathological Parameters *n* = 107		OS (CI 95%)	*p* Value
Sex	Male	44 (16.66–71.34) 44 (10.63–77.37)	0.96
	Female		
Age	<65 years	60-month OS 51.4% 60-month OS 42.5%	0.10
	≥65 years		
Cardiac disease	Yes	36 (15.20–56.80) 47 (22.56–71.44)	0.53
	No		
Clinical stage (cTNM) *	I–II	81 (57.46–104.53) 26 (12.91–39.09)	<0.01
	III–IV		
Tumor anatomical location	GEJA vs. Gastric, Others	19 (0–54.65) 18 (11.83–24.16)	0.86
Histological WHO classification	Tubular/Papillary	48 (21.36–74.64) 43 (3.69–82.3)	0.63
	Others (Mixes, Mucinous, Poorly Cohesive)		
Histological Laurén classification	Intestinal vs. Diffuse	12-month OS 74.6% vs. 53.8%	0.93
	Diffuse vs. Indeterminate type	12-month OS 53.8% vs. 75.0%	0.39
	Intestinal vs. Indeterminate type	12-month OS 74.6% vs. 75.0%	0.23
Growth pattern	Infiltrative	12-month OS 88.7% 12-month OS 45.0%	0.37
	Other		
Depth of invasion (T)	T1 + T2	81 (56.07–105.92) 28 (13.33–42.67)	0.02
	T3 + T4a		
Lymphatic permeation	Present	42-month OS 42.1% 42-month OS 49.4%	0.01
	Absent		
Perineural invasion	Present	36-month OS 24.4% 36-month OS 51.2%	<0.01
	Absent		
Vascular invasion	Present	34 (20.19–47.80) 79 (37.37–120.63)	0.07
	Absent		
Surgical margins—R0	Yes	48 (12.14–83.89) 23 (4.66–41.34)	0.02
	No		
Lymph node metastasis (pN)	Present	48-month OS 20.4% 48-month OS 47.7%	<0.01
	Absent		
Distant metastasis after surgery (pM)	Present	16 (5.15–26.85) 61 (29.28–92.72)	<0.01
	Absent		
Pathological stage (pTNM) *	I–II	88 (66.37–109.62) 24 (14.47–33.53)	<0.01
	III–IV		
Upfront treatment approach	ESD or Surgery	61 (23.19–98.81) 33 (14.45–51.54)	0.11
	POCT or NACRT		
NA treatment (*n* = 31)	POCT	23 (3.78–42.20) vs. 47 (28.60–59.40)	0.88
	NACRT		
POCT/NACRT conclusion (*n* = 31)	Yes	66 (13.66–118.34) 19 (10.68–27.32)	0.03
	No		
Tumor response grade after POCT (*n* = 31) **	1–2	28 (10.32–45.67) 63 (8.72–117.28)	0.31
	3		
ACT (*n* = 76 ***)	Yes	18-month OS 55.0% 18-month OS 69.4%	0.10
	No		
ACT conclusion (*n* = 22)	Yes	24-month OS 87.5% 24-month OS 75.0%	0.37
	No		
HER2	Negative	36-month OS 57.7% 36-month OS 33.3%	0.32
	Positive		
PD-L1 CPS (*n* = 11)	<1 vs. 1–5	21-month OS 50.0% vs. 75.0%	0.46
	<1 vs. ≥5	21-month OS 50.0% vs. 40.0%	0.89
	1–5 vs. ≥5	21-month OS 75.0% vs. 40.0%	0.42

Legend: ACT: adjuvant chemotherapy, CPS: combined positive score, ESD: endoscopic submucosal dissection, GEJA: gastroesophageal junction adenocarcinoma, NACRT: neoadjuvant chemoradiotherapy, POCT: perioperative chemotherapy, WHO: World Health Organization. * 8th edition American Joint Committee on Cancer, ** Becker Classification, *** number of patient eligible to ACT (excluded those who did POCT and NACRT).

**Table 6:** Association between clinicopathological/therapy features and overall survival (OS) (multivariate analysis).

Clinicopathological Parameters *n* = 107		Multivariate Analysis
		Hazard Ratio, (CI 95%), *p*-Value
Clinical stage *	I–II vs. III–IV	0.77, (0.20–3.02), *p* = 0.71
Depth of invasion (pT)	T1 + 2 vs. T3 + T4a	0.35, (0.06–2.00), *p* = 0.24
Perineural invasion	Present vs. Absent	0.60, (0.17–2.16), *p* = 0.44
Surgical margins—R0	Yes vs. No	1.59, (0.28–8.83), *p* = 0.60
Lymph node metastases (pN)	Present vs. Absent	0.06, (0.01–0.48), *p* = 0.01
Distant metastases after surgery (pM)	Present vs. Absent	0.87, (0.26–2.89), *p* = 0.82
Pathological stage *	I–II vs. III–IV	2.57, (0.44–14.95), *p* = 0.29
POCT/NACRT conclusion	Yes vs. No	0.58, (0.17–1.91), *p* = 0.37

Legend: NACRT: neoadjuvant chemoradiotherapy, POCT: perioperative chemotherapy. * 8th edition American Joint Committee on Cancer.

## Data Availability

All data concerning this work are included in the manuscript. Data supporting the findings of this study are available, upon reasonable request, from the corresponding author (H.O.D). The data are not publicly available due to privacy restrictions (they contain information that could compromise the privacy of research participants).
